# A review of safety risk management and optimization strategies for physical education classes in Chinese schools in heat-stress environments

**DOI:** 10.3389/fpubh.2026.1803928

**Published:** 2026-04-16

**Authors:** Jiaxin Li, Yiwu Yang, Mingang Guo, Jingfei Wang

**Affiliations:** 1 School of Physical Education, Wuhan University of Technology, Wuhan, China; 2 Hubei Research Center for the Inheritance and Innovation of Ethnic Traditional Sports Culture, Wuhan, China; 3 Physical Education Department, Northwestern Polytechnical University, Xi An, China

**Keywords:** global warming, heat acclimatization, outdoor activity, physical education, physical exertion

## Abstract

Global warming influences rising temperatures, making outdoor physical education classes progressively challenging to organize. While heat acclimatization is essential for athletic development, the conventional emphasis on “endurance” training in Chinese schools sometimes overlook the physical limitations of youths. This paper reviews outdoor sports in heat acclimatization related published articles and examines the body’s response to heat stress and evaluates whether physical education professionals are prepared to reduce these risks. This review found that many coaches and teachers lack the requisite practical knowledge to prevent heat stress, often reacting to issues only post-occurrence; a comparison of international standards highlights a further issue: unlike other countries, which follows strict guidelines, China lacks clear and specific guidelines for youth sports safety in heat acclimatization; Heat stress significantly affects physical performance and willingness to participate in activities among youths and heat acclimatization training is an effective intervention method. To address this, we recommend using the student military training period as an opportunity to build heat acclimatization. Additionally, physical education classes must be updated to prioritize hydration, proper clothing, and controlled exercise intensity during hot weather. Establishing these standard safety rules is essential to protect students and ensure they can continue participating in sports safely.

## 1 Introduction

There is a broad consensus in the field that physical education classes provide a critical, structured environment for fostering consistent physical activity in students (1). Guided by the ecological behavior model, contemporary research emphasize that environmental variables—specifically extreme heat stress—act as decisive factors in physical activity participation (2); furthermore, the specific spatial context of physical education has been identified as a critical determinant of student engagement and safety (3). Physical education class in China has distinct characteristics compared to those in other countries (4). Chinese physical education classes are typically characterized by big group [more than 65% of classes have >30 students (5)] and limited indoor facilities, which necessitates a reliance on outdoor instruction. Over the past two decades, physical education classes globally have undergone significant transformations (5–7). Within Chinese higher education, physical education remains a mandatory component of the curriculum (8). Within these physical education classes, a majority of students are engaged in the comprehensive compulsory education curriculum reform initiated by the Chinese Ministry of Education in April 2022. This policy framework emphasizes the improvement of students’ comprehensive physical and mental health while promoting healthy lives. Central to this initiative is the integration of traditional Chinese martial arts—specifically Tai Chi and Changquan—and indigenous folk sports, such as dragon boat racing, into the established outdoor athletic program comprising football, basketball, tennis, and track and field (9).

The Urban Heat Island (UHI) phenomenon caused with worldwide urban expansion, resulting in unique microclimatic difficulties in metropolitan areas (10). This phenomenon compounds local and regional warming trends, creating a synergistic effect with the escalating frequency and intensity of global heatwaves. Thus, it contributes to the heat vulnerability of urban populations and presents a substantial impediment to the realization of sustainable urban development goals (11). Many regions across China-most notably the “Four Furnaces” (Chongqing, Wuhan, Nanjing, and Nanchang)-experience extreme heat conditions during the academic calendar, with heat temperatures frequently recorded during the late spring and early autumn transitions (12, 13). Empirical data from 2021 suggests that peak physiological performance is achieved within temperate, low-humidity environments, with the optimal heat window identified at approximately 10–15 °C (14, 15). Conversely, exposure to high-temperature conditions induces significant heat stress, thereby decreasing aerobic capacity and overall physical activity levels. While outdoor environments provide the spatial capacity necessary for large-scale student cohorts, the escalating frequency of extreme weather events attributed to global climate change necessitates a re-evaluation of scheduling guidelines. Specifically, the management of outdoor physical education during periods of intense summer heat has emerged as a critical point of scholarly and administrative discourse. The rising number of extreme heat events imposes significant restricts on the institutional capacity to facilitate safe and effective physical education. Consequently, balancing the requirement for student physical activity with the necessity of reducing heat-related health risks has become an increasingly complex logistical and pedagogical challenge. Physical effort under these settings may cause excessive sweating, increasing the risk of severe dehydration. Hypohydration can precipitate significant physiological disruptions, particularly electrolyte imbalances. Current literature indicates that such deficits result in marked impairments across aerobic capacity, muscular endurance, and cognitive functioning (16–19). While the progression of heat-related illnesses is both debilitating and subtle, these conditions are fundamentally preventable through systematic monitoring and intervention. It is imperative that both educators and students cultivate a comprehensive understanding of symptomology to facilitate proactive measures in safeguarding physical well-being. Developmental differences exist regarding physiological self-regulation; generally possess a more acute awareness of their hydration state than younger students (20). In addition, it is critical to recognize that physiological predispositions, specifically elevated adiposity and pre-existing chronic comorbidities, significantly increased susceptibility to heat dehydration. In these vulnerable group, fluid deficits frequently manifest as acute fatigue and physiological discomfort, often accompanied by psychological stressors such as heightened irritability and reduced cognitive focus (5, 21). Despite the growing urgency of the climate crisis, empirical investigations into heat-related impacts and heat awareness remain limited, with existing research primarily concentrate on specific groups such as sports tourists and recreational endurance athletes (22). Consequently, there is a significant lack of research addressing these dynamics within the context of general student populations or compulsory educational settings (23). Due to current climate changes, conducting outdoor physical education during seasonal heat peaks has become a significant difficulty for both educators and students. The existing pedagogical framework lacks standardized guidelines for adapting to high-temperature environments. Consequently, educators have a binary decision: to suspend scheduled sessions, which compromises curricular continuity, or to maintain routine operations, which increases students’ health risks. This study examines adaptive management strategies for the 90-min instructional block and evaluates optimized training frameworks aimed at reducing heat-related morbidity in outdoor environments.

## 2 Method

The search strategy for this review was developed in accordance with the PRISMA (Preferred Reporting Items for Systematic Reviews and Meta-Analyses) Extension for Scoping Reviews (PRISMA-ScR) guidelines. A systematic investigation of extant research article was conducted via structured and exploratory queries across several prominent bibliographic databases, including the Web of Science (Core Collection), PubMed (National Library of Medicine), MEDLINE, and SPORTDiscus. These databases were selected for their rigorous indexing standards and extensive coverage of peer-reviewed research, providing a robust and empirically valid basis for this review. Eligibility Criteria Studies were included if they met the following PCC (Population, Concept and Context) criteria: (1) Individuals participating in outdoor sports or classes. (2) Impact of heat exposure or high temperature on physical activity. (3) Outdoor environments during heat acclimatization or summer.

### 2.1 Search strategy and selection criteria

To ensure comprehensive coverage, a manual search of the reference lists from the selected studies was performed to identify additional relative literature. The final search was executed on October 1, 2025. The specific search method and keyword combinations are listed in Table 1. Initial screening was conducted through a rigorous evaluation of titles and abstracts to determine eligibility for full-text review.

**Table 1:** Searching strategy.

Search strategy	Description
#1	Hot temperature/seasons
#2	Environment/controlled
#3	Sports/exercise
#4	#1 AND # 2 AND # 3

The literature search utilized a combination of Boolean descriptors and truncated terms, including: “Hot Temperature,” “Sports” OR “Exercise,” “Heat Stress Disorders,” “Physical Education and Training” and “Hot Temperature.” To be eligible for inclusion in this review, studies were required to meet the following stringent criteria: (a) empirical focus on high-temperature environments, with the exclusion of aquatic sports; (b) participation in physical activity or athletic activity, irrespective of demographic variables such as age, gender, or proficiency; (c) publication within peer-reviewed scholarly journals; and (d) dissemination in the English language. No temporal restrictions were placed on the year of publication. Conversely, studies were excluded if they: (a) focused on occupational manual labor; (b) targeted specific athletic disciplines without the general “outdoor” descriptor; or (c) were categorized as conference abstracts or review articles.

### 2.2 Data extraction and quality assessment

**Figure 1:**
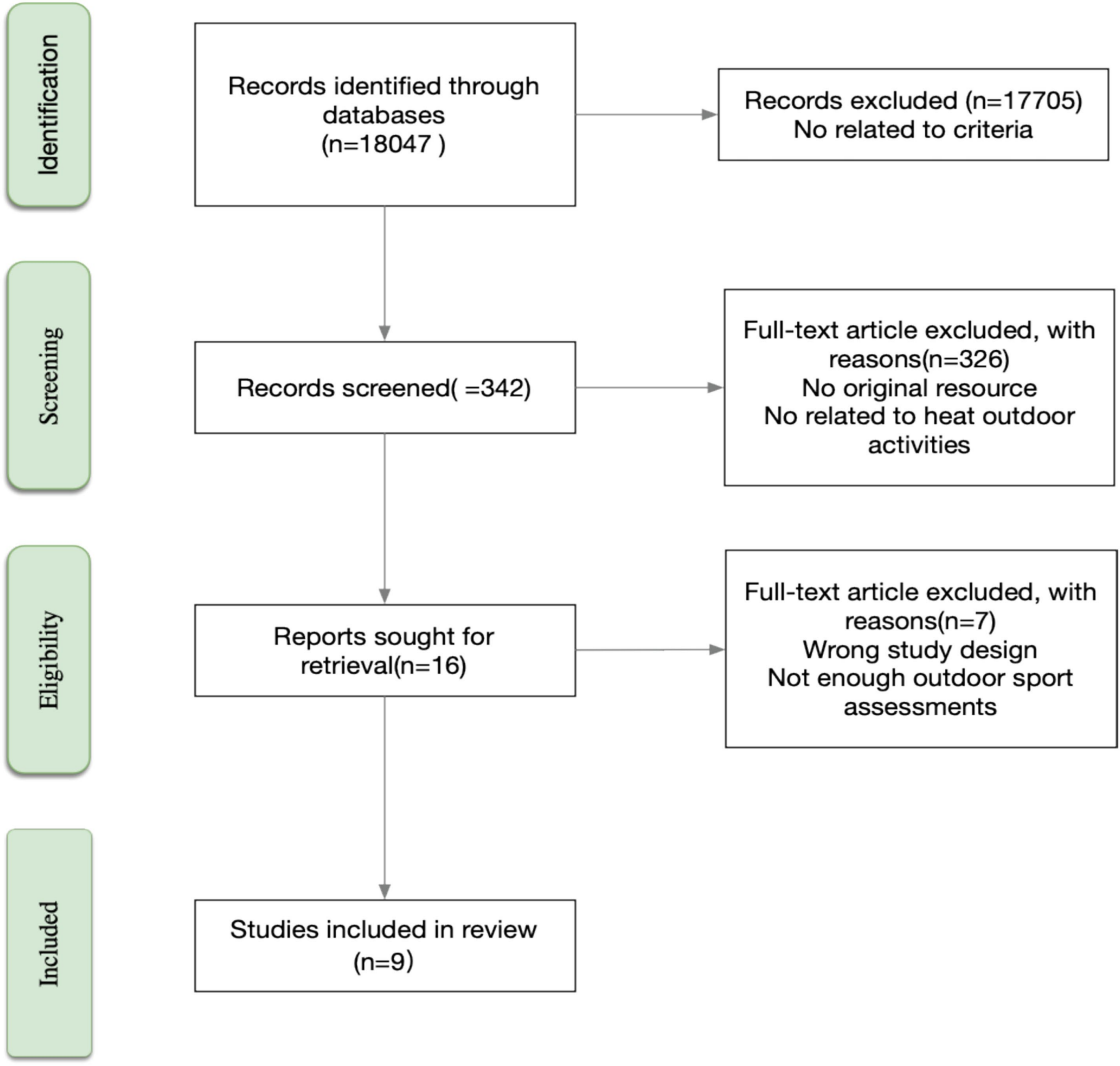
Methodology of selected articles.

The initial search produced 18,047 potentially relevant records. To reduce bias and ensure methodological integrity, two senior investigators with extensive expertise in heat physiology and sports performance independently evaluated each article for relevance and quality. Following a multi-stage screening process governed by the pre-defined inclusion and exclusion criteria, 9 articles were identified as meeting all requirements for qualitative and quantitative synthesis. The selection trajectory is detailed in the PRISMA flow diagram (Figure 1).

## 3 Results

Four comprehensive tables summarize the principal conclusions of the review. The nine studies detailed in Table 2 are geographically diverse. They include five European studies, two from Australia, and one each from Singapore and the United States. According to the absence of relevant literature and the exclusion of irrelevant research, no age-related limits were imposed, which allowed for a more comprehensive data synthesis.

**Table 2:** Participates information.

Study	Country	Published date	Subjects	Sample size	Gender	Age	Body mass (kg)	Height (cm)	V.O_2_max (mL kg^−1^ min^−1^)
Daniel et al. (32)	Australia	3-Sep-18	Team sport athletes (Australian football or soccer)	16	Males	20.9 ± 1.9; 24.5 ± 3.6	80.36 ± 7.40; 82.04 ± 9.95	180.9 ± 6.4; 182.1 ± 8.8	54.3 ± 2.5; 56.3 ± 3.6
Leer et al. (29)	Germany	4-Dec-24	Outdoor sports coaches	1,200	Children: 26% adolescents: 40%; adults: 34%.	44 ± 14	–	–	–
Byrne et al. (26)	Singapore	20-Nov-18	Competitive half-marathon males	24	Males	26 ± 3	65.5 ± 6.5	172 ± 5	59 ± 5
Wallenberg et al. (27)	Sweden	19-Sep-23	Preschool Children	9	Five girls and four boys	5-year-old	16.6; 15.7	115.9; 119.0	–
Del Coso et al. (28)	Spain	13-May-14	Well-trained triathletes	34	30 males and 4 females	36 ± 6	73.6 ± 6.4	178 ± 7	–
Sambrook et al. (30)	United Kingdom	16-May-23	Elite athletes from 10 different sports (including race walking, netball and cricket)	14	5 females; 9 males	20–41	–	–	–
Pryor et al. (24)	United States	19-Jun-18	Non-heat acclimatized men	16	Males	–	–	–	CON: 57.3; IHE: 55.2
Özgünen et al. (54)	Turkey	28-Apr-10	Football player	11	Males	20.4 ± 2.1	68.5 ± 5.3	176.8 ± 4.8	62.6 ± 6.8
Brown et al. (25)	Australia	16-Oct-23	Recreationally active adults	17	7 females; 10 males	28 ± 8	71.1 ± 10.8	180 ± 10	54 ± 8

A significant difference occurs between qualitative and quantitative methods concerning participant groups. The survey data encompassed almost 1,200 respondents; however, the experimental treatments often comprised smaller sample sizes (*n* ≈ 20), predominantly comprising male volunteers. The demographic range was broad, including persons from 5 to 42 years of age. Of the nine selected papers, eight included age and gender metrics; however, three did not give complete anthropometric or physiological data, such as body mass (kg), height (cm), and V̇O2max (mL kg^−1^ min^−1^).

Table 3 provides a comprehensive systematic analysis of the experimental designs and research methodology utilized in the chosen studies. The thematic focus was divided: seven articles examined physiological exertion and athletic performance in hyperthermic conditions, while two utilized qualitative frameworks to assess the perspectives and adaptive strategies of elite athletes and coaching staff in relation to climate change. The qualitative research utilized semi-structured interviews and questionnaires, producing cross-sectional data points. Conversely, the other seven empirical investigations utilized longitudinal or repeated-measures designs, integrating multiple data collecting intervals for all participants. In addition to foundational anthropometric variables (height, mass, and pulmonary capacity), the studies utilized diverse instrumentation to monitor systemic physiological responses. These included the quantification of core thermometry (via intestinal sensors), heart rate variability, fluid dynamics (ad libitum intake and urinary output) and hematological parameters.

**Table 3:** Study design.

Study	Study design	Aim	Estimating method	Subjects assessment
Daniel et al. (32)	A pair-matched, between subjects design	To evaluate the effects of continuous (CON) and intermittent (INT) heat acclimatization regimens on repeat-sprint performance and to measure the extent of performance deterioration post-acclimatization.	8 times	Participants performed a heat tolerance test (HTT) consisting of 60 min of repeated sprint cycling, interspersed with a 10-min halftime break, conducted 2 days prior to the HTT and following acclimatization (post-HTT1). Decay was examined through two more HTTs conducted over the subsequent 2 weeks (post-HTT2 and post-HTT3).
Leer et al. (29)	An asynchronous online survey conducted by computer-aided web interviewing (CAWI) utilizing a standardized questionnaire and the Lime Survey software package.	The increase in global temperatures is increasing the risk of heat-related illnesses in outdoor sports. Coaches are obligated to protect the athletes under their supervision. This study assessed the knowledge and practices of German coaches on heat prevention on a national scale.	From May 2022 to June 2023	Online survey: individual interview, email and phone call questionnaire
Byrne et al. (26)	Observational study	The objective is to evaluate whether the physiological strain index (PSI), in its original or modified version, can effectively measure heat strain on a 0–10 scale among trained and heat-acclimatized men participating in a competitive half-marathon in outdoor heat conditions.	Twice	Four weeks before to the event, all participants conducted an incremental treadmill test to volitional exhaustion to ascertain VO_2_peak and maximal heart rate. During the race, TC and HR were recorded at 15-s intervals and averaged across one-minute periods. Pre-race hydration status measures (including urine specific gravity) and fluid balance were evaluated as previously outlined. Environmental parameters were assessed during the race.
Wallenberg et al. (27)	Measurement and observations of children’s activity	This study aims to examine the impact of warm weather on the heat comfort and physical activity of 5-year-old children in a preschool yard in Sweden.	8 times	Collection proceeded for 1–1.5 h in the early afternoon (1–3 p.m.) and was done over 8 days in May, June, and August of 2022. Polar Team Pro sensors; GPS tracker.
Del Coso et al. (28)	Experimental	This study aimed to evaluate physiological factors before and after a half-ironman triathlon and to analyze their correlation with performance. We hypothesized that reductions in body mass, hypoglycemia, electrolyte imbalance, and muscle injury would correlate positively with diminished performance in a half-Ironman triathlon.	Twice	Three hours before to the race, competitors congregated at a location near the start line without any suggestions regarding pre-exercise hydration or nutrition. Telemetry pill for the assessment of intestinal temperature (HT150002, HQ Inc., US). A 10-min warm-up was conducted; participants executed two countermovement vertical jumps (CMJs) for maximum height on a force platform (Quattro-jump, Kistler, Switzerland) to evaluate pre-race leg power output.
Sambrook et al. (30)	Qualitative approach	Investigated professional athletes’ views, experiences, and reactions to excessive heat in the context of climate change, and analyzed their potential for climate activism through their platforms.	Once	Semi-structured interviews
Pryor et al. (24)	Randomized control trial	To ascertain if intermittent exercise-heat exposures (IHE) every fifth day maintain heat acclimatization (HA) adaptations 25 days post-initial HA.	Every fifth day for 25 days	Participants were pair-matched based on self-reported physical activity and peak oxygen consumption (VO_2_peak) measurements and body surface area to regulate parameters recognized to influence thermoregulation and probable HA degradation.
Özgünen et al. (54)	Control trial	This study assessed the activity patterns and temperature reactions of players during soccer matches conducted under varying environmental conditions.	Twice	Temperature, maximum oxygen (VO_2_max); heart rate; thermosensor, water, urine and weight
Brown et al. (25)	Longitudinal, repeated measures design.	To determine the extent of seasonal heat acclimatization in recreationally active people and to contextualize the process by recording the factors that affect adaptations.	Initial (pre-summer) testing began in November/December,	Questionnaires; VO_2_max; blood test, sweat; To assess the effects of seasonal heat acclimatization on resting Toes and HR, the change in Toes and HR during a heat response test, Tsk, LSR, sweat [Na^+^], WBSR and percent body mass loss,

Table 4 shows that seven of the nine included studies employed diverse experimental methodologies to provide an empirical quantification of physiological responses across various demographic cohorts under hyperthermic conditions. Conversely, two investigations (Sophie and Kate) utilized qualitative inquiries—specifically semi-structured interviews—to explore the nuanced perspectives of outdoor sports stakeholders, such as organizers and coaches, regarding risk perception and precautionary training guidelines.

**Table 4:** Article type.

Adaptive programming	Physiological performance	Coaches and teachers knowledge to protect heat stress
Daniel et al. (32)	Byrne et al. (26)	Leer et al. (29)
Pryor et al. (24)	Wallenberg et al. (27)	Sambrook et al. (30)
Brown et al. (25)	Del Coso et al. (28)	–
–	Özgünen et al. (54)	–

An analytical synthesis of the existing literature identifies three principal thematic domains: adaptive programming, physiological performance, and health literacy alongside emergency intervention, as showed in the conceptual framework below:

Resent research find that heat acclimatization protocol (HAP) implemented prior to formal training can preserve individuals performance during subsequent temperate and humidity condition exercise, humidity increasing affect sweat to evaporate, causing heat to build up in the body, though these benefits appear restricted to a specific temporal window. Given that current analyses are predominantly restricted to the relationship between heat gain and heat loss elements, they fail to account for the influence of confounding training variables; consequently, it remains undetermined whether these adaptive shifts are driven by enhancements in aerobic capacity. Table 5 shows the main findings in detail. Supplementary findings by Pryor et al. (24) further demonstrate that intermittent heat exposure significantly mitigates both perceived exertion and objective physiological strain during exercise-induced heat stress. Furthermore, longitudinal data from Brown et al. (25) suggest that while outdoor exercise typically facilitates seasonal heat acclimatization, the process is highly sensitive to environmental fluctuations. Specifically, anomalous climatic events—such as La Niña—may impede the development of these adaptive responses, leading researchers to question the extent to which ambient temperature variability, rather than cumulative exercise duration, dictates the overall efficacy of heat acclimatization.

**Table 5:** Main finding.

Study	Test environment	Outcome measures	Overall findings	Limitation
Daniel et al. (32)	35.3 ± 0.7 °C, 60.1 ± 4.0% relative humidity	No differences in final core and mean skin temperatures or heart rate existed after INT or CON acclimatization, however 30 min measures for heat sensation, perceived thirst and ratings of perceived exertion (as well as the final measure) were lower in post-HTT1 (*p* < 0.05) in CON. Performance and thermoregulatory responses in post-HTT2 and 3 were similar to post-HTT1 in both INT and CON.	The results demonstrate that repeated repeat-sprint exercise in heat is enhanced following acclimatization through brief, high-intensity cycling sessions with either CON or INT guidelines, with performance sustained over the following 2 weeks, despite the absence of the heat stimulus.	Although participants were matched for aerobic fitness levels and body surface area (ratio of heat gain and heat loss elements), further matching based on thermoregulatory responses to pre-HTT could have enhanced the comparison between groups. The absence of a control group hinders the ability to differentiate between acclimatization and training effects in any observed performance changes. Ultimately, conducting the multi-stage fitness test, as utilized during familiarization, at the conclusion of the acclimatization period would have facilitated the assessment of any changes in aerobic fitness.
Leer et al. (29)	Over 30 °C	The KOSI produced an average score of 10.31 ± 1.81, indicating significant knowledge deficits. Coaches in skiing (9.85 ± 1.80), soccer (10.07 ± 2.33), and golf (10.09 ± 1.75) recorded the lowest scores, with a *p*-ANOVA value of 0.015. Heat protection during training was inadequate, with the HPS indicating a mean value of 62.41 ± 14.89. The most significant deficits were observed in tennis (57.71 ± 14.29), mountain sports (58.17 ± 13.08), and soccer (58.70 ± 13.86; *p*-ANOVA <0.001). No correlation was identified between theoretical knowledge and practical prevention.	In Germany, coaches lack adequate preparation for the health risks associated with heat exposure. Promoting onsite educational programs is crucial for ensuring safer sports environments.	This study has several weaknesses, including potential selection biases, fundamental critiques of the official recommendations, and limitations regarding its applicability to other regions.
Byrne et al. (26)	Averaged 26.4 °C and 81% relative humidity.	In a warm (26.1–27.3 °C) and humid (79–82%) environment, all runners completed the race asymptomatically in an average time of 107 ± 10 (91–137) minutes. The peak temperature and heart rate were 39.7 °C ± 0.5 (range: 38.5–40.7 °C) and 186 ± 6 (range: 175–196) beats per minute, respectively. Sixty-three percent surpassed a temperature of 39.5 °C, 71% exceeded a heart rate of 180 beats per minute, and 50% exceeded both the original upper constraints for temperature and heart rate as defined by the PSI.	The PSI was able to quantify heat strain on a 0–10 scale in trained and heat acclimatized men undertaking a half-marathon race in outdoor heat, but only when the upper TC and HR constraints were modified to 41.0 °C and age-predicted maximal HR, respectively.	This study indicates that a significant proportion of PSI responses is expected to exceed the value of 10. The 0–10 scale is a significant strength of PSI, and it is recommended that this feature be preserved through straightforward modifications of PSI constraints tailored to the specific population under investigation.
Wallenberg et al. (27)	15.1°C–26.4 °C	The findings indicate that physical activity diminishes in warmer weather conditions, as evidenced by reductions in distance traveled, step counts, and maximum recorded heart rate. Additionally, during warm days, children tend to avoid sunlit areas.	This study highlights the significance of shaded areas in preschool yards, enabling children to engage in active play while preserving a safe heat condition.	Measurement sensors have not been utilized in five-year-old children.
Del Coso et al. (28)	29 ± 3 °C	The mean race time was 315 ± 40 min, with swimming accounting for 11 ± 1%, cycling for 49 ± 2%, and running for 40 ± 3% of the total race duration. At the conclusion of the competition, body mass exhibited a change of −3.8 ± 1.6%, with a positive correlation observed between the change in body mass and race time (*r* = 0.64; *p* < 0.001). Core temperature rose from 37.5 ± 0.6 °C to 38.8 ± 0.7 °C (*p* < 0.001), and post-race core temperature exhibited a negative correlation with race time (*r* = −0.47; *p* = 0.007). The correlation between race time and the reduction in jump height was positive (*r* = 0.38; *p* = 0.043), as well as with post-race serum creatine kinase levels (*r* = 0.55; *p* = 0.001) and myoglobin concentrations (*r* = 0.39; *p* = 0.022).	In a half-Ironman triathlon conducted in high temperatures, faster triathletes exhibited more significant reductions in body mass and elevated post-competition core temperatures. Conversely, slower triathletes exhibited elevated muscle damage and diminished muscle performance.	–
Sambrook et al. (30)	Online in English interview	Four broad topics include: health and performance; care and concern for sport and society; implications of heatwave experiences; and enablers and barriers to effective climate change communication.	Sport organizations must prioritize educating athletes, coaches, and event organizers about the effects of heat on sport participation as they confront the challenges posed by climate change. This increased knowledge and awareness aim to mitigate illness among individuals engaged in training and competition.	The snowballing approach, along with the lack of multiple coders, may compromise reliability.
Pryor et al. (24)	IHE; *n* = 9, 40 °C, 40% RH; (CON; *n* = 7, 24 °C, 21% RH)	Both groups exhibited similar heat acclimatization, as indicated by reduced heart rate and thermoregulatory, physiological, and perceptual responses, including perceived exertion, fatigue, and heat sensation. Pre-HA versus Post-HA.	Exercise and heat exposures every fifth day for 25 days, along with regular intense physical activity, resulted in sustained heart rate and core temperature adaptations, as well as reduced perceptual and physiological strain during exercise heat stress approximately 1 month later.	–
Özgünen et al. (54)	34 ± 11 °C; relative humidity 38 ± 2%	In soccer matches conducted under elevated ambient temperatures and relative humidity, players exhibit an increase in body core temperature by halftime, accompanied by a reduction in total distance covered during the second half. This phenomenon may indicate a centrally driven decline in performance.	In soccer matches conducted under elevated environmental temperature and humidity, players’ physical performance may decline as a result of increased heat stress.	–
Brown et al. (25)	40 °C and 30% relative humidity	The increase in oesophageal temperature and mean skin temperature during the heat response test was comparatively lower. Additionally, the local sweat rate in the forearm exhibited an increase. Minimal evidence was observed regarding changes in heart rate or whole-body sweat rate during the heat response test.	Evidence suggests that recreationally active adults exhibited partial heat acclimatization following summer. However, the combination of exercising later in the day and the prevailing environmental conditions, specifically La Niña in Southeastern Australia, may have hindered the progression of additional adaptations.	The uncharacteristically temperate conditions during the summer of testing likely hindered the increase in WBSR in the current study, rather than the amount of outdoor exposure among participants.

Substantial evidence suggests that heat stress exerts a deleterious impact on both physiological function and physical performance. Research by Byrne et al. (26) demonstrated that while participants successfully performed tasks under thermoneutral conditions, exposure to an ambient temperature of 38 °C caused core body temperatures to exceed 39.5 °C in approximately half the group, a response concomitant with significantly rising heart rates. Similarly, Wallenberg et al. (27) observed that in five-year-old children, hot weather suppressed spontaneous physical activity, leading to marked reductions in travel distance, step count, and peak heart rate.

However, endurance athletes display divergent physiological responses depending on performance level. Del Coso et al. (28) found that elite half-ironman triathletes tolerated higher core temperatures and greater weight loss, while slower athletes suffered disproportionately from muscle damage and functional decline despite lower heat loads. Evidence from professional soccer further corroborates the limiting role of heat; hyperthermia in humid conditions activates central nervous system inhibition, resulting in a measurable attenuation of performance.

There remains a significant gap between the recognition of heat stress risks and the implementation of effective prevention strategies within the sports community. Using the KOSI (heat-related illness symptoms index) instrument, Leer et al. (29) demonstrated that outdoor coaches, particularly in Germany, lacked the necessary theoretical foundation and preparedness to address high-temperature hazards. Complementing this, Sambrook et al. (30) examined the awareness profiles of event organizers and participants, identifying similar shortcomings. These findings point to an urgent need for comprehensive educational strategies. Disseminating scientific evidence regarding heat stress is essential for equipping stakeholders with the knowledge to proactively manage risk and reduce the prevalence of heat-related diseases.

## 4 Discussion

This review categorizes the nine studies into three distinct thematic clusters: (a) adaptive training under heat stress; (b) the competency of outdoor sports coaches and educational staff and (c) in-situ performance analysis in hot conditions. Consequently, practical strategies are proposed to address the challenges identified within these areas.

On the first theme, Fortney et al. (31) posited that high-temperature environments accelerate the elevation of both core and cutaneous temperatures, thereby predisposing individuals to heat-induced injury in 1985. Consequently, they formulated a set of guidelines designed to reduce heat stress during physical activity in hot conditions (31). Daniel et al. (32) demonstrated that while pre-cooling strategies serve to preserve peak power in non-acclimatized individuals, physiological heat acclimatization is necessary to actively improve power output (33). Exposure to these severe environmental constraints exacerbates the susceptibility to exertional heat illness; consequences include deficits in motor coordination (34) and cognitive processing (35), alongside general performance impairment (36). In urgent situations, this thermal stress may result in heatstroke and fainting (37). Insufficient rest, hydration, and nutritional intake, combined with a lack of heat acclimatization, significantly compromise the heat tolerance of youth, thereby elevating their susceptibility to exercise-induced heat illness (38). The feasibility of implementing heat acclimatization guidelines within standard physical education class warrants critical evaluation. Effective acclimatization necessitates a gradual, incremental stimulation, typically requiring continuous daily exposure. This presents a significant logistical challenge for university physical education programs, which are often restricted to weekly sessions (e.g., two 45-min blocks). However, freshman military training, frequently executed in conditions of high ambient temperature, presents a feasible option. Implementing acclimatization strategies at the beginning of this intensive training period could effectively reduce the risk of heatstroke among the students.

In organized sports, coaches serve as the principal guardians of athlete safety, a responsibility that spans competitive tiers and age demographics. The structural environment of coaching varies considerably between countries. In Germany, where school-based physical education is limited to approximately 2 h per week—similar to the class in China (34)—athletic development is largely centralized in local clubs, often supervised by coaches holding only part-time credentials. Conversely, youth sports in the United States are primarily organized within schools and overseen by professional coaching personnel (35, 36).

On the second theme, this structural divergence extends to heat safety guidelines. Different countries have specific rules to protect students during summer heatwaves. Unlike professional athletes, students are more vulnerable to heat, so schools must follow strict safety steps. In Australia, heat management is managed by individual states. Their policies are very strict about “SunSmart” habits. Most schools use a “no hat, no play” rule to ensure students stay protected from the sun while outdoors (39). As a tropical nation, Singapore uses the Wet Bulb Globe Temperature (WBGT) index to measure heat and humidity. When the index is high, teachers must give students mandatory water breaks or move all activities into indoor, shaded areas (40–42). To deal with rising temperatures caused by climate change, Spain updated its laws in 2023. If the national weather agency issues a Red or Orange alert, outdoor physical activity classes are completely banned. During the summer, many schools also schedule PE as the very first class of the day to avoid the afternoon sun (43). The UK Health Security Agency provides specific advice for schools. If there is an Amber or Red heat alert, outdoor physical activities must be replaced with indoor ones. Teachers and students are also told to wear loose, light-colored clothes and reapply sunscreen every 2 h (44). While Sweden is known for cold winters, it now faces high summer temperatures as well. The government has started issuing specific heat safety advice to help the general public and schools manage these unusual heatwaves (45). The United States has established specific pre-season heat acclimatization guidelines for secondary schools (37), emphasizing critical, albeit debated, therapeutic treatments for exertional heatstroke: rectal thermometry and cold water immersion (38). Also require a gradual implementation of equipment, including limitations on helmets and pads. In terms of supervision, outdoor activities in high temperatures for students (especially school team athletes) are strictly regulated by school sports associations at all levels, the Centers for Disease Control and Prevention (CDC) (46); National Athletic Trainers’ Association (NATA) (47), and National Federation of State High School Associations (NFHS) (48). In contrast, China’s strategy is less specialized; existing documentation mostly provides generalized public health advisories for heatwaves, devoid of focused advice for student athletics or specific curricula. The German guidelines prioritize prophylactic, whereas the US emphasizes acute clinical management. In accordance with worldwide standards, German guidelines emphasize preventive measures with reduced intervention thresholds, encompassing scheduling modifications, weight-adaptive hydration, equipment alterations, and the compulsory presence of medical personnel (6, 7, 11). Through a comparison of the three countries’ approaches to deal with high-temperature environments problem, in the United States, heat protection for students has evolved from mere “recommendations” to stringent industry standards. The U.S. employs data and legal regulations for strict oversight. If schools fail to follow NFHS guidelines, resulting in student deaths from heatstroke, they face substantial compensation claims. Thus, the guidelines carry strong “risk-avoidance” attributes and are implemented rigidly and thoroughly. Germany places greater emphasis on self-discipline and the professional qualifications of coaches, with an insurance system differing from that of the U.S., relying more on social welfare safeguards. China lacks a unified, detailed mandatory standard linking temperature to exercise duration, depending more on administrative decisions by schools. Culturally, unlike the other two countries, China has long upheld the rigorous notion of “training in the coldest winter days and the hottest summer days,” sometimes viewing endurance under high temperatures as a test of willpower, which may create a gray area between scientific protection and willpower training.

The majority of preventive strategies outlined here require minimal logistical effort in terms of time and resources. Hydration strategies—before, during, and after training—should be established as standard practice rather of being dependent exclusively on high temperatures. Likewise, exterior cooling measures typically necessitate only basic materials, such water buckets, towels, and reusable ice packs, making them both economical and time-efficient. Nevertheless, findings from the second article indicate a disparity between availability and execution; these straightforward instructions were not uniformly adopted throughout training sessions. This compliance gap may arise from a reactive mentality, when athletes regard prophylaxis as superfluous in the absence of explicit indications of heatstroke.

On the third theme, in this context, Article 6 (3) highlighted significant disparities in health literacy regarding heat stress, varying by sport, resource access, and athletic level. Even elite athletes frequently demonstrate ambiguity regarding the urgency of climate-related risks. The harmful consequences of heat are pervasive, impacting athletes of all ages and fitness levels. Proactive preparation is more prevalent among those with a previous history of heatstroke or performance deficits. Considering the variability in individual thermoregulatory capacity, dependence on heat tolerance may unintentionally distort team selection, so undermining sports equity by prioritizing physiological resilience above technical talent.

Regarding performance physiology, Article 3 (26) reported that one-third of acclimatized athletes exceeded core temperatures of 40 °C during physical activity. Fatigue in well-trained subjects typically begins at this critical threshold (49), although theoretical lethal limit for humans approximating 42 °C (50). Pediatric populations are also vulnerable; Article 4 (27) demonstrated that environment temperatures above 20 °C suppress physical activity in children (51), evidenced by reduced motility and lower peak heart rates (52). While dehydration is a known meaningful impairer of endurance (37), core temperature elevation is primarily driven by metabolic heat production. To prevent catastrophic failure, the brain engages in anticipatory regulation. As proposed by Tucker et al. (53), the central nervous system responds to high rates of heat storage by down-regulating work rate—manifesting as reduced speed or intensity—to limit further heat accumulation and prevent biological damage.

In considering these findings for the context of physical education in China, several recommendations are proposed. First, based on the evidence that “heat adaptation requires continuous stimulation,” there are practical difficulties in achieving heat acclimatization with only 1–2 physical education classes per week in China. The freshman military training period should be strategically utilized to integrate heat acclimatization, thereby enhancing physiological readiness. Second, physical education class during hot seasons must explicitly address the pathology of heatstroke, emphasizing its potential to compromise function and endanger life. It is recommended to develop evidence-based and graded (e.g., temperature-humidity index) safety guidelines for school physical activities. Instructors incorporate thermal safety knowledge into teacher training and develop emergency plans, and also should enforce hydration plan that include electrolytes (sodium) and carbohydrates for extended activity. Furthermore, internal cooling strategies, such as the ingestion of cold fluids before and during exercise, are recommended to expand heat storage capacity. Finally, clothing selections should prioritize evaporative cooling, and while sedentary behavior is not recommended, untrained students should be prescribed low-intensity, short-duration activities in shaded environments to facilitate gradual adaptation. In this review, it also has limitations. For example, the inclusion of most researches are adults, lack of adolescent samples; the sample size is small and has regional bias. The focus of future research should be on conducting intervention studies on heat acclimatization among Chinese adolescents.

## 5 Conclusion

Current research on heat acclimatization and athlete cognizance reveal a distinct disparity: while the risks of high temperatures are understood in theory, sports professionals often fail to prevent them in practice. A major strength in this study is the recognition that only “hearing information” about heatstroke is not enough. In this study, highlighting that simple measures (hydration, cooling) are often overlooked in daily training, the study identifies a specific failure point in public health implementation. Even though the dangers of heatstroke are well known, simple and effective measures like drinking enough water, using external cooling, and gradual heat acclimatization are frequently overlooked in training. It advocates for teaching students and teachers how to distinguish between “normal discomfort” and “dangerous physical stress.”

To address this in China, the student military training period offers a valuable opportunity to introduce heat acclimatization training on a large scale. At the same time, physical education classes in hot regions need to update their teaching to focus on “heat safety,” helping students and teachers tell the difference between normal discomfort and dangerous physical stress. Future guidelines should emphasize the development of specialized, practical guidelines for youth sports rather than just relying on general public health advice. By coordinating administrative regulations with the physiological requirements of the body, we can protect student health and ensure sports participation continues safely in a warming climate.

While the study identifies a “disparity,” it also has limitation. It does not provide specific data on “how often” these ignores happen or the specific incidence rates of heat illness in these settings. This makes it harder to estimate the serious problem. The study may underestimate the difficulty of changing rigid school schedules or military training protocols, which is a common barrier in public health policy.
